# microRNA regulators of apoptosis in cancer

**DOI:** 10.15190/d.2016.4

**Published:** 2016-04-01

**Authors:** Octavian Bucur

**Affiliations:** Department of Pathology, Harvard Medical School and Beth Israel Deaconess Medical Center, Boston, MA, USA

**Keywords:** miRNAs, non-coding RNAs, apoptosis dysregulation, evasion of apoptosis, malignancies

## Abstract

This brief review summarizes our current knowledge on the microRNAs that regulate apoptosis machinery and are potentially involved in the dysregulation or deregulation of apoptosis, a well known hallmark of cancer. microRNAs are critical regulators of the most important cellular processes, including apoptosis. Expression of microRNAs is found to be dysregulated in many malignancies, leading to apoptosis inhibition in cancer, or resistance to current therapies. To date, there are over 80 microRNAs directly involved in apoptosis regulation or dysregulation that can impact cancer detection, initiation, progression, invasion, metastasis or resistance to anti-cancer therapy. Development of microRNA-based therapeutic strategies is now taking shape in the clinic. Thus, these microRNAs represent potential targets or tools for cancer therapy in the future.

## **1. **Introduction

**Figure 1:**
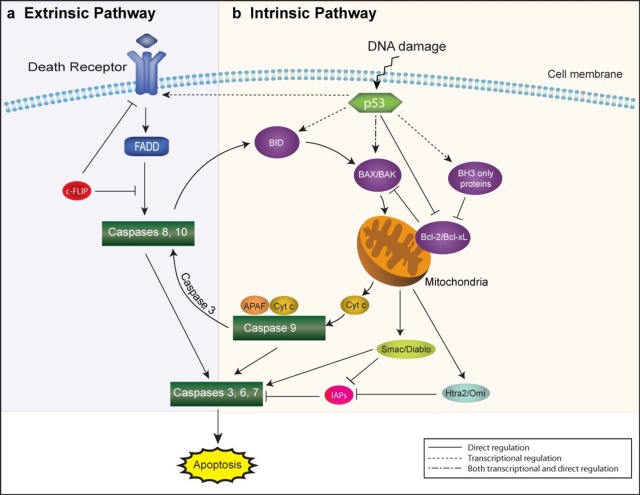
Figure 1. Extrinsic (receptor-mediated) and intrinsic (mitochondria-mediated) pathways of apoptosis cooperate in activating effector caspases (3,6 and 7) and inducing apoptosis Most of the components of these pathways can be targeted/regulated by microRNAs, including, but not limited to the ligands, death receptors, p53, Bcl-2 family members (Bim, Bax, Bcl-2, Bcl-xL, Mcl-1), XIAP and caspases.

Apoptosis is an evolutionary conserved physiological form of cell death required for removal of harmful and unuseful cells^1-4^. Apoptosis is not only necessary for the development and maintenance of the cellular and organismal homeostasis^1-4^, but it also serves as a defense mechanism, removing cells infected by viruses^5^. Signaling pathways that lead to cell death by apoptosis have as central effectors the proteolytic enzymes names caspases^1-4,6^. Caspases, which are cysteine-aspartic proteases or cysteine-dependent aspartate-directed proteases, are important not only in executing apoptosis, but are also involved in executing other forms of cell death (caspase-dependent cell death) and in inflammation^1-4,7^. General consensus established two pathways of apoptosis: the extrinsic pathway, which is mediated by death receptors, and the intrinsic pathway, which is mediated by mitochondria^1-4^. Apoptosis dysregulation is a hallmark of cancer and many studies focused on targeting these mechanisms as an anti-cancer strategy^1-4^. A schematic representation of the apoptotic pathways is presented in**Figure 1.**

Over the last decade, microRNAs have emerged as critical regulators of normal and cancer cell structure and functions. microRNAas are involved in the regulation and control of most, if not all, cellular responses and fate. Some of these fundamental cellular processes that are linked to cancer and tumorigenesis are apoptosis, cell proliferation, stress response, differentiation and development^8-13^.In the last decade, a significant number of studies revealed and demonstrated without doubt the involvement of the microRNAs and other non-coding RNAs in tumorigenesis, invasion, metastasis and susceptibility to anti-cancer therapies^8-13^. Moreover, regulation of apoptosis by microRNAs is one of the important mechanisms that can lead to apoptosis inhibition in cancer or resistance to current cancer therapies^8,9,13^.

MicroRNAs are small, 19-22 nucleotides long, non-coding RNAs, that negatively regulate gene expression, by translation inhibition and stepping up the degradation of the specific target mRNAs^8,9^. There have been more than few thousands microRNAs identified until now, which have been found to regulate more than 60% of the genes from human genome^14^. A single microRNA can regulate many (maybe even hundreds of) transcripts and a single transcript can have binding sites for (or can be regulated by) several microRNAs of the same or different sequence^8,9,14^. Currently, the major methods used to discover and verify the microRNAs and other non-coding RNAs are RNAseq and qPCR. While qPCR remains the standard for verification, RNAseq is starting to be widely employed in the microRNA identification^15^.

This brief review summarizes the most important microRNAs involved in apoptosis regulation or dysregulation in cancer.

## 2. microRNA regulators of apoptosis in cancer (summary in Table 1)

Dysregulated microRNA expression has been observed in cancer tumorigenesis and this process is at least in part explained by epigenetic silencing of microRNA genes^12^. In the recent years, significant progress was made in the identification and characterization of the microRNAs involved in targeting genes critical for apoptosis process, including genes involved in apoptotic pathways or the regulatory pathways of apoptosis. The expression of many of these microRNAs is found to be dysregulated in cancers^12^, inducing an altered expression of the target genes and resulting in dysregulated apoptosis.

As an example, miR-125b targets p53 gene expression, decreasing p53-induced apoptosis in neuroblastoma cells^16^. p53 is not only a target of microRNAs, but can also regulate, similar to other transcription factors (Myc and E2Fs^11^) the microRNAs expression. p53 directly regulates the expression of miR-34 family members (miR-34a, b, c), which mediates p53-induced apoptosis, and miR-34 downregualtion correlates with p53 mutation/downregulation and resistance to p53 induced apoptosis in pancreatic cancer^17^, lung cancer^18^, or neuroblastoma^19^. Noteworthy, other microRNAs are involved in either directly targeting the expression of death receptors or their ligands, or in modulating sensitivity to death ligands/ non-ligand-induced apoptosis. For example, miR-25 targets TNF-related apoptosis inducing ligand (TRAIL) death receptor-4 and promotes apoptosis resistance in cholangiocarcinoma^20^. Moreover, miR-221 and miR-222 are implicated in modulating the sensitivity to Apo2L/Trail-induced apoptosis in lung cancer cells, partly by direct targeting p27kit1 or Kit genes. These two microRNAs were also shown to directly target the estrogen receptor alpha (ER-alpha) and to confer resistance to Tamoxifen induced apoptosis in breast cancer^21,22^ (see**Table 1**).

Most (if not all) Bcl-2 family members were shown to be direct targets of microRNAs, with altered expression in different types of cancers. For example, Mcl-1 (which is an anti-apoptotic member) is targeted by miR-29 and miR-113B (cholangiocarcinoma, lung cancer)^23,24^. Bim (a pro-apoptotic member) is targeted by miR-17-92 (B cell lymphoma)^25^ and Bmf (a pro-apoptotic member) is a direct target of miR-221 (hepatocellular carcinoma)^26^. Many other microRNAs were shown to target these Bcl-2 family members, as well as other members of the family, such as Bax and Bcl-xL (see**Table 1**).

Caspases, the central effectors of apoptosis, are also confirmed targets of several microRNAs, including microRNA-224, involved in lung cancer pathogenesis, by targeting caspases 3 and 7^27^. Other microRNAs targeting caspases are summarized in**Table 1**.

Other genes involved in apoptosis regulation are also targeted by microRNAs, but are not discussed in this review. This long list includes the transcription factors such as Myc^28^ and FOXOs^29^, phosphatases such as PTEN or PP2A, or kinases such as PI3K, Akt, Erk, Mek (and other kiases related to MAPK pathways)^30-32^.

Moreover, anti-cancer therapeutic strategies based on the regulation of microRNA activity have promise as anti-cancer treatments, since microRNAs have the ability to modulate fundamental cellular processes, such as apoptosis, cell proliferation or differentiation^8,9^. Systemic administration of microRNAs in mice (adeno viruses-mediated or by other strategies) successfully induced tumor-specific apoptosis. Noteworthy, no significant toxicity was observed^33^.

## 3. Conclusions

This brief review highlights our up-to-date basic knowledge on the microRNAs that regulate apoptosis machinery and are potentially involved in its dysregulation/deregulation, a well defined hallmark of cancer.

In cancer, many microRNAs can act as either oncogenes or tumor suppressors, and their action is context dependent^8,9^. Thus, many of the microRNAs mentioned in this article that regulate apoptosis can potentially be classified in one of these two categories.

microRNAs are involved in targeting expression of genes involved in apoptosis/cell survival, proliferation, differentiation, tumorigenesis, tumor invasion, angiogenesis and more. Expression of certain microRNAs is responsible for resistance of different malignancies to at least some of the current employed cancer treatments. Moreover, expression of specific microRNAs can predict patient outcomes. Regulation of apoptosis by microRNAs is one of the important mechanisms that can lead to apoptosis inhibition in cancer, or resistance to current therapies. Our current knowledge reveals over 80 microRNAs involved in apoptosis regulation or dysregulation, that can impact cancer initiation, progression, invasion, metastasis or resistance to anti-cancer therapy (**Table 1**). Moreover, circulating microRNA biomarkers are investigated for detection of tumor cells, diagnosis of different pathologies and for evaluation of treatment response.

microRNA abnormalities were identified in many types of malignancies and other diseases, and the development of miRNA-based gene therapy is now taking shape in clinical practice^8,9^.

**Table 1:** TABLE 1. microRNAs regulating apoptosis pathways in cancer

Target Gene	microRNA	Comments	Ref.
Death Receptors & Ligands	miR-20a	encoded by the miR-17-92 cluster increases the metastatic potential of osteosarcoma cells by regulating Fas expression	^34^
	miR-21	targets Fas ligand-mediated apoptosis in breast cancer cell line MCF-7	^35^
	miR-23a/b	up-regulated expression of miR-23a/b targeted the pro-apoptotic Fas in radiation-induced thymic lymphoma	^36^
	miR-25	targets TNF-related apoptosis inducing ligand (TRAIL) death receptor-4 and promotes apoptosis resistance in cholangiocarcinoma	^20^
	miR-34	CD95 is part of a let-7/p53/miR-34 regulatory network	^37^
	miR-106a	is frequently upregulated in gastric cancer and inhibits the extrinsic apoptotic pathway by targeting FAS	^38^
	miR-467a	is upregulated in radiation-induced mouse thymic lymphomas and regulates apoptosis by targeting Fas and Bax	^39^
			
Bim	miR-17	inhibition of miR-17 by oridonin triggers apoptosis and reverses chemoresistance by derepressing BIMs	^40^
	miR-17-5p	miR-17-5p inhibitor enhances chemosensitivity to gemcitabine via upregulating Bim expression in pancreatic cancer cells	^41^
	miR-20	controls the mitochondrial apoptotic machinery by fine-tuning the levels of expression of the proapoptotic protein BIM	^42^
	miR-25	regulates apoptosis by targeting Bim in human ovarian cancer.	^43^
	miR-92	controls the mitochondrial apoptotic machinery by fine-tuning the levels of expression of the proapoptotic protein BIM	^42^
	miR-106b-25	miR-106b-25 polycistron, activated by genomic amplification, functions as an oncogene by suppressing p21 and Bim.	^44^
	miR-124	Regulates Apoptosis and Autophagy Process in MPTP Model of Parkinson's Disease by Targeting to Bim	^45^
	miRNA-148a	is a prognostic oncomiR that targets MIG6 and BIM to regulate EGFR and apoptosis in glioblastoma; promotes Th1-cell survival by regulating the proapoptotic gene Bim.	^46^
	miR-181	targets the 3'UTRs of Bcl-2 family members Bcl-2-L11/Bim, Mcl-1, and Bcl-2	^47^
	MiR-192	suppresses apoptosis and promotes proliferation in esophageal aquamous cell caicinoma by targeting Bim	^48^
	miR-302	control the mitochondrial apoptotic machinery by fine-tuning the levels of expression of the proapoptotic protein BIM	^42^
	miR-363	promotes human glioblastoma stem cell survival via direct inhibition of caspase 3, caspase 9, and Bim.	^49^
	miR-494	is regulated by ERK1/2 and modulates TRAIL-induced apoptosis in non-small-cell lung cancer through BIM down-regulation.	^50^
	miR-582-5p	promotes human glioblastoma stem cell survival via direct inhibition of caspase 3, caspase 9, and Bim.	^49^
			
Bax	miR-128	downregulates Bax and induces apoptosis in human embryonic kidney cells; downregulation of miRNA-128 sensitises breast cancer cell to chemotheray by targeting Bax	^51,52^
	miR-298	bufalin promotes apoptosis of gastric cancer by down-regulation of miR-298 targeting Bax	^53^
	miR-467a	is upregulated in radiation-induced mouse thymic lymphomas and regulates apoptosis by targeting Fas and Bax	^54^
			
Bmf	mRNA-221	targets Bmf in hepatocellular carcinoma and correlates with tumor multifocality.	^26^
			
Bcl-2	miR 15/15a	induce apoptosis by targeting BCL2; induce the apoptosis of rat activated pancreatic stellate cells by targeting Bcl-2 in vitro; modulate multidrug resistance by targeting BCL2 in human gastric cancer cells; miR-15b mediates liver cancer cells proliferation through targeting BCL-2	^55-58^
	miR 16/16-1	induce apoptosis by targeting BCL2; induce the apoptosis of rat activated pancreatic stellate cells by targeting Bcl-2 in vitro; modulate multidrug resistance by targeting BCL2 in human gastric cancer cells; inhibits glioma cell growth and invasion through suppression of BCL2 and the nuclear factor-κB1/MMP9 signaling pathway	^55-57,59^
	miR-21	inhibitor suppresses proliferation and migration of nasopharyngeal carcinoma cells through down-regulation of BCL2 expression	^60^
	miR-24-2	miR-195, miR-24-2 and miR-365-2 act as negative regulators of BCL2 through direct binding to their respective binding sites in the 3'-UTR of the human BCL2 gene (J Cell Sci 2012)	^61^
	miR-30b	functions as a tumour suppressor in human colorectal cancer by targeting KRAS, PIK3CD and BCL2	^62^
	miR-34a	Prognostic significance of miR-34a and its target proteins of FOXP1, p53, and BCL2 in gastric MALT lymphoma and DLBCL	^63^
	miR-125a-5p	inhibits cell proliferation and induces apoptosis in colon cancer via targeting BCL2, BCL2L12 and MCL1	^64^
	miR-181b	modulates multidrug resistance by targeting BCL2 in human cancer cell lines.	^65^
	miR-182	Role of microRNA-182 in posterior uveal melanoma: regulation of tumor development through MITF, BCL2 and cyclin D2.	^66^
	miR-184	Tumor suppressor PDCD4 modulates miR-184-mediated direct suppression of C-MYC and BCL2 blocking cell growth and survival in nasopharyngeal carcinoma.	^67^
	miR-195	miR-195, miR-24-2 and miR-365-2 act as negative regulators of BCL2 through direct binding to their respective binding sites in the 3'-UTR of the human BCL2 gene (J Cell Sci 2012)	^61^
	mR-204	Transformer 2β and miR-204 regulate apoptosis through competitive binding to 3' UTR of BCL2 mRNA.	^68^
	mR-205	A novel regulator of the anti-apoptotic protein Bcl2, is downregulated in prostate cancer	^69^
	mR-206	Down-regulation of c-Met and Bcl2 by microRNA-206, activates apoptosis, and inhibits tumor cell proliferation, migration and colony formation	^70^
	miR-210	MicroRNA-210 targets antiapoptotic Bcl-2 expression and mediates hypoxia-induced apoptosis of neuroblastoma cell	^71^
	miR-365-2	miR-195, miR-24-2 and miR-365-2 act as negative regulators of BCL2 through direct binding to their respective binding sites in the 3'-UTR of the human BCL2 gene (J Cell Sci 2012)	^61^
	miR-449a	miR-449a Regulates proliferation and chemosensitivity to cisplatin by targeting cyclin D1 and BCL2 in SGC7901 cells.	^72^
	miR-503	miR-503 regulates cisplatin resistance of human gastric cancer cell lines by targeting IGF1R and BCL2.	^73^
	miR-1271	miR-1271 regulates cisplatin resistance of human gastric cancer cell lines by targeting IGF1R, IRS1, mTOR, and BCL2.	^74^
	miR-1290	miRNA-1290 promotes asiatic acid induced apoptosis by decreasing BCL2 protein level in A549 non small cell lung carcinoma cells.	^75^
			
Bcl-xL	Let-7	The let-7 family of microRNAs inhibits Bcl-xL expression and potentiates sorafenib-induced apoptosis in human hepatocellular carcinoma; Lin28 inhibited let-7 family microRNA levels and upregulated the anti-apoptotic protein Bcl-xL, which is a target of let-7; Let-7c sensitizes acquired cisplatin-resistant A549 cells by targeting ABCC2 and Bcl-xL	^76-78^
	miR-34a	MicroRNA-608 and microRNA-34a regulate chordoma malignancy by targeting EGFR, Bcl-xL and MET	^79^
	miR-133a	miRNA-133a, downregulated in osteosarcoma, suppresses proliferation and promotes apoptosis by targeting Bcl-xL and Mcl-1.	^80^
	miR-491	miR-491-5p targeting both TP53 and Bcl-xL induces cell apoptosis in SW1990 pancreatic cancer cells through mitochondria mediated pathway; miR-491-5p-induced apoptosis in ovarian carcinoma depends on the direct inhibition of both BCL-xL and EGFR, leading to BIM activation.	^81,82^
	miR-574-3p	Genistein up-regulates tumor suppressor microRNA-574-3p in prostate cancer: miR-574-3p restoration induced apoptosis through reducing Bcl-xL and activating caspase-9 and caspase-3	^83^
	miR-608	MicroRNA-608 and microRNA-34a regulate chordoma malignancy by targeting EGFR, Bcl-xL and MET	^84^
			
Mcl-1	miR-26a	miR-26a inhibits proliferation and migration of breast cancer through repression of MCL-1	^85^
	miR-29	miR-29a down-regulation in ALK-positive anaplastic large cell lymphomas contributes to apoptosis blockade through MCL-1 overexpression; Overexpression of microRNA-29b induces apoptosis of multiple myeloma cells through down regulating Mcl-1; Exosome-derived microRNA-29c induces apoptosis of BIU-87 cells by down regulating BCL-2 and MCL-1	^23,86-88^
	miR-101	MicroRNA-101 targets EZH2, MCL-1 and FOS to suppress proliferation, invasion and stem cell-like phenotype of aggressive endometrial cancer cells; MicroRNA-101 inhibits cell progression and increases paclitaxel sensitivity by suppressing MCL-1 expression in human triple-negative breast cancer.	^89,90^
	miR-125a-5p	miR-125a-5p inhibits cell proliferation and induces apoptosis in colon cancer via targeting BCL2, BCL2L12 and MCL1	^64^
	miR-125b	miRNA-125b promotes apoptosis by regulating the expression of Mcl-1, Bcl-w and IL-6R	^91^
	miR-106a	MiR-106a targets Mcl-1 to suppress cisplatin resistance of ovarian cancer A2780 cells	^92^
	miR-133	MicroRNA-133a, downregulated in osteosarcoma, suppresses proliferation and promotes apoptosis by targeting Bcl-xL and Mcl-1; miRNA-133b targets pro-survival molecules MCL-1 and BCL2L2 in lung cancer.	^24,93^
	miR-181b	miR-181b increases drug sensitivity in acute myeloid leukemia via targeting HMGB1 and Mcl-1.	^94^
	miR-205	miR-205 and miR-218 expression is associated with carboplatin chemoresistance and regulation of apoptosis via Mcl-1 and Survivin in lung cancer cells	^95^
	miR-218	miR-205 and miR-218 expression is associated with carboplatin chemoresistance and regulation of apoptosis via Mcl-1 and Survivin in lung cancer cells	^95^
	miR-193	Ionizing radiation-inducible microRNA miR-193a-3p induces apoptosis by directly targeting Mcl-1; miR-193b regulates Mcl-1 in Melanoma; miR-193b Modulates Resistance to Doxorubicin in Human Breast Cancer Cells by Downregulating MCL-1.	^96-98^
	miR-302b	miRNA-302b Enhances the Sensitivity of Hepatocellular Carcinoma Cell Lines to 5-FU via Targeting Mcl-1 and DPYD	^99^
	miR-320	Down-regulation of miR-320 associated with cancer progression and cell apoptosis via targeting Mcl-1 in cervical cancer.	^100^
	miR-363	miR-363 sensitizes cisplatin-induced apoptosis targeting in Mcl-1 in breast cancer.	^101^
			
XIAP	miR-24	miRNA-24 regulates XIAP to reduce the apoptosis threshold in cancer cells	^102^
	miR-130a	Downregulation of miR-130a contributes to cisplatin resistance in ovarian cancer cells by targeting X-linked inhibitor of apoptosis (XIAP) directly	^103^
	miR-192-5p	Curcumin promotes apoptosis by activating the p53-miR-192-5p/215-XIAP pathway in non-small cell lung cancer	^104^
	miR-200	miR-200bc/429 cluster modulates multidrug resistance of human cancer cell lines by targeting Bcl-2 and XIAP; microRNA-200c downregulates XIAP expression to suppress proliferation and promote apoptosis of triple-negative breast cancer cells.	^105,106^
	miR-215	Curcumin promotes apoptosis by activating the p53-miR-192-5p/215-XIAP pathway in non-small cell lung cancer	^104^
	miR-429	miR-429 mediates δ-tocotrienol-induced apoptosis in triple-negative breast cancer cells by targeting XIAP; miR-200bc/429 cluster modulates multidrug resistance of human cancer cell lines by targeting Bcl-2 and XIAP	^105,107^
	miR-519	miR-519d represses ovarian cancer cell proliferation and enhances cisplatin-mediated cytotoxicity in vitro by targeting XIAP	^108^
	miR-618	miR-618 inhibits anaplastic thyroid cancer by repressing XIAP in one ATC cell line	^109^
			
Caspases	miR-221	Expression patterns of miR-221 and its target Caspase-3 in different cancer cell lines;	^110^
	miR-224	MicroRNA-224 is implicated in lung cancer pathogenesis through targeting caspase-3 and caspase-7	^27^
	miR-421	MiR-421 regulates apoptosis of BGC-823 gastric cancer cells by targeting caspase-3	^111^
	miR-574	Genistein up-regulates tumor suppressor microRNA-574-3p in prostate cancer: miR-574-3p restoration induced apoptosis through reducing Bcl-xL and activating caspase-9 and caspase-3	^112^
			
p53	miR-19b	miR-19b promotes tumor growth and metastasis via targeting TP53.	^113^
	miR-96	MicroRNA-96 promotes the proliferation of colorectal cancer cells and targets tumor protein p53 inducible nuclear protein 1, forkhead box protein O1 (FOXO1) and FOXO3, known inhibitors of Bim, p21, p27115	^114,115^
	miR-214	microRNA-214 enhances the invasion ability of breast cancer cells by targeting p53	^116^
	miR-300	miR-300 regulate the malignancy of breast cancer by targeting p53	^117^
	miR-491-5p	miR-491-5p targeting both TP53 and Bcl-XL induces cell apoptosis in SW1990 pancreatic cancer cells through mitochondria mediated pathway.	^81^
